# Management of Functional Disorders of Stomach

**Published:** 1889-09

**Authors:** P. R. Cortelyou

**Affiliations:** Marietta, Ga.


### ON THE MANAGEMENT OF FUNCTIONAL DISORDERS OF THE STOMACH.

Read before the Georgia State Medical Association, at Macon, Ga., April 19, 1889.

BY P. R. CORTELYOU, A. M., M. D., MARIETTA, GA.

In calling the attention of this Association for a short time to some points "On the Management of Functional Disorders of the Stomach," I am fully aware that the subject is trite, and that nothing especially new can be said in regard to it. That it has been thoroughly written on by able and scholarly minds, and that in a manner so fully that it would be hard indeed to add even a few threads of purely original thought. My apology, if one be necessary, must be found in this: that it is not the rare and infrequent diseases that most often perplex the daily practitioner, but the more common maladies of every day life, and those which often are considered to be of little special interest, yet it is often in these very cases that our patients expect and look for speedy relief.

It is not my purpose, nor is it necessary at this time that we should discuss the physiology of gastric digestion, nor the importance of its proper performance, for the securing of good health. Suffice it to say that the stomach is one of the most important, as well as perhaps the most abused organs in the human system. Some ancient solon has said, "Keep the head cool, the feet warm, and the stomach all right, and you will have little need of doctors." However that may be, we know that the condition of the stomach is at the foundation of many diseases.

When we consider what the human stomach has to endure, in receiving into it things hot and cold, raw, boiled, fried, and at all times and hours, from early morn often until the midnight hour, we cannot wonder that it will fail at times to do the work placed upon it.

We are told that Sampson, with the jaw-bone of an ass, slew a thousand men, but we think that the hot biscuit and frying pan have slew their tens of thousands.

Among the causes affecting the digestion may be mentioned predisposing and exciting causes. Everything which causes depressed vitality is a predisposing cause to indigestion. It may be caused by hot and debilitating climate, changes in the elementary constituents of the blood, exhausting diseases, mental and moral emotions act as predisposing causes. Age, also, in the extremes of old age and infancy. Anæmia, and deficiency of the gastric juice, are also predisposing causes. The immediate exciting causes are errors in diet, excessive eating and drinking. The use of unwholesome food and such as is not properly prepared. Too frequent introduction of food into the stomach without giving the organ sufficient time for rest, acts as an exciting cause. Also irregularity in eating, and rapid eating, the food not being thoroughly masticated.

In treating these cases all these things must be considered and overcome to effect a cure. In the following reported cases, which have been under my care during the past year, I have adopted a somewhat routine line of treatment, but one that has proven very beneficial, and therefore I report them at this time.

Case 1. Miss H., single, æt. 35 years. I was called to see the patient October 3, 1887. She gave me the following history. She had been suffering for months with severe pain in the stomach, and severe vomiting, often unable to sleep at night on account of the pain. She had lost a good deal of flesh, and was unable to sit up all day. Could eat no solid food without having pain and distress. There was marked tenderness over the epigastrium. The patient very despondent and apprehensive of cancer. Had used various remedies without receiving benefit. Ordered glass of hot water to be taken one hour before each meal, and pill consisting of arsenious acid $\frac{1}{30}$ grain, extract nux vomica $\frac{1}{6}$ grain, belladonna $\frac{1}{6}$ grain, reduced iron 1 grain, to be taken after each meal. Blister, size of silver dollar, over epigastrium, and powder of ingluvin 10 grains, before each meal. Diet, milk, some beef essence, boiled rice. October 6. Patient improving, less pain after eating, sleeps better at night. Ordered bismuth subnit. 4 grains, pulv. pepsin 4 grains, pulv. ipecac gr. $\frac{1}{3}$ before each meal, in place of ingluvin. The pills and hot water continued. October 10. Patient still improving. Bowels costive. Ordered fld. ext. cascara sag. 20 to 30 drops at night. I increased diet, giving stale light bread, crackers, soft boiled egg. Patient able to get out of doors when pleasant. From this date she steadily improved, until she fully regained her strength and was able to take regular diet, her friends telling her she had not looked so well for years, and she still continues to keep well.

Case 2. Judge C., æt. 71. Called to see the patient April 22, 1888. Gave following history: He had suffered from chronic bronchial trouble for years, and has been spending the winters in Georgia and Florida. He had just come from Florida and was suffering with severe cough, just recovering from an acute attack of bronchitis. His stomach was very tender on pressure, and he was unable to take food without pain and distress, and nausea. Ordered quieting mixture for the cough. Glass of hot water one hour before each meal, with elix. lactopeptine after each meal, and small blister over the epigastrium. Diet: milk, beef essence, crackers, and stale bread. Patient improved rapidly under this treatment, the hot water having a very beneficial effect on his stomach. In about ten days he was able to take regular diet with appetite and without distress.

Case 3. Miss B., æt. 40. Called to see patient July 1, 1888. She gave following history: For several weeks had been suffering with severe pain and distress on taking food, and with severe choking spells. Complains of burning and boring pains in the stomach, and tenderness on pressure. Much emaciated, unable to sleep, pale and nervous, anæmic and despondent. Had been under treatment, but received no benefit. Ordered glass of hot water one hour before each meal, small blister over the epigastrium, and powder of bismuth and pepsin, 5 grains each, after meals; aromat. spts. of ammonia, Hoffman's anodyne, and spts. lavender co., for choking spells. Diet:

milk and lime water, stale bread and some chicken broth. July 9th. Patient improving, but still complaining of pain in the stomach, but not so constant. Ordered powder of $\frac{1}{7}$ gr. calomel, $\frac{1}{15}$ gr. morph. sulph., 5 grains bismuth subnit., after each meal. Patient improved steadily on this treatment with the hot water; increased diet, giving soft boiled eggs, soft boiled rice, tea, soda crackers. July 31, 1888. Patient gaining in strength and color and flesh, has very little pain after eating. Ordered pill of $\frac{1}{30}$ grain arsenious acid, $\frac{1}{5}$ grain ext. nux vomica, and 1 grain each of quinine and reduced iron, after each meal. From this date patient steadily improved, and returned to her regular diet, and had no further trouble.

Case 4. Mrs. M. colored, æt. 35. I was consulted by patient July 31, 1888. She gave the following history: Had been for weeks suffering with severe pain in stomach and left side; unable to take food without pain. Had been under treatment, but received no benefit. Ordered glass of hot water one hour before each meal, fly blister over stomach, powder of bismuth and pepsin, 4 grains each, after meals. Diet: milk and lime water, stale bread, and soft boiled rice. Patient improved rapidly on this line of treatment. August 9th. Patient able to take food without pain or distress. Ordered mixture of nux vomica, bismuth and carbolic acid, after each meal, and from this time she has had no further need of treatment.

Case 5. Mr. A., æt. 35, married. Called to see patient November 29, 1888. He gave the following history: For about one year had been suffering from severe attacks of pain in stomach and bowels, which unfitted him from attending to his business, and frequently prevented sleep. Had lost considerable flesh and was very despondent. Had been under various lines of treatment, but received no permanent relief. His skin was sallow, tongue coated with white fur, bowels costive, tenderness over the epigastrium and liver. Ordered glass of hot water one hour before each meal, small blister over the epigastrium, mild laxative for constipation, and mixture of acid nitro hydrochl., dil. ʒiss, syr. sarsaparilla co., ℥ij; one teaspoonful to be taken after each meal. Diet: milk and lime water, beef essence, stale bread and soft boiled rice. On this line of treatment patient continued to improve and was relieved of pain and distress, gradually became able to take fuller diet, and gained in strength and flesh. April 1, 1889. The patient has been at work during the winter, has gained 15 pounds in flesh and able to take his regular meals without trouble, but is still using the hot water once a day and the nitro. hydchl. pil. occasionally.

From the results obtained in these cases, with others not reported. I have been led to feel that in hot water properly used, we have a very beneficial agent in all catarrhal conditions of the stomach, and one that is generally grateful to the patient. I have also found the use of small fly blister over the stomach to aid in relieving nausea and pain and tenderness, and think that these means, together with proper regulation of diet, will be successful in relieving many of these distressing and troublesome disorders of the stomach, which are functional.

